# Customized Mobile LiDAR System for Manhole Cover Detection and Identification

**DOI:** 10.3390/s19102422

**Published:** 2019-05-27

**Authors:** Zhanying Wei, Mengmeng Yang, Liuzhao Wang, Hao Ma, Xuexia Chen, Ruofei Zhong

**Affiliations:** 1 Beijing Advanced Innovation Center for Imaging Theory and Technology, Capital Normal University, Beijing 100048, China; zhanyingwei@sina.com (Z.W.); mh8036101@126.com (H.M.); xuex_chen@126.com (X.C.); zrfsss@163.com (R.Z.); 2 Chinese Academy of Surveying and Mapping, Beijing 100830, China; jzwlzcf@sohu.com; 3 State Key Laboratory of Automotive Safety and Energy, Department of Automotive Engineering, Tsinghua University, Beijing 100084, China; 4 Center for Intelligent Connected Vehicles and Transportation, Tsinghua University, Beijing 100084, China

**Keywords:** mobile mapping system, manhole cover, detection, identification, assessment

## Abstract

Manhole covers, which are a key element of urban infrastructure management, have a direct impact on travel safety. At present, there is no automatic, safe, and efficient system specially used for the intelligent detection, identification, and assessment of manhole covers. In this work, we developed an automatic detection, identification, and assessment system for manhole covers. First, we developed a sequential exposure system via the addition of multiple cameras in a symmetrical arrangement to realize the joint acquisition of high-precision laser data and ultra-high-resolution ground images. Second, we proposed an improved histogram of an oriented gradient with symmetry features and a support vector machine method to detect manhole covers effectively and accurately, by using the intensity images and ground orthophotos that are derived from the laser points and images, respectively, and apply the graph segmentation and statistical analysis to achieve the detection, identification, and assessment of manhole covers. Qualitative and quantitative analyses are performed using large experimental datasets that were acquired with the modified manhole-cover detection system. The detected results yield an average accuracy of 96.18%, completeness of 94.27%, and F-measure value of 95.22% in manhole cover detection. Defective manhole-cover monitoring and manhole-cover ownership information are achieved from these detection results. The results not only provide strong support for road administration works, such as data acquisition, manhole cover inquiry and inspection, and statistical analysis of resources, but also demonstrate the feasibility and effectiveness of the proposed method, which reduces the risk involved in performing manual inspections, improves the manhole-cover detection accuracy, and serves as a powerful tool in intelligent road administration.

## 1. Introduction

An increasing number of urban manhole covers are being used in road administration (e.g., for electricity and gas supply, fire control, water supply and draining, communication, and sewage discharge) as rapid urban development in the digital age promotes constant growth, city planning improvements, and road construction. The daily management, maintenance, and inspection of these covers now involve increased workloads and difficulties. Frequent and heavy traffic also leads to cover damage and subsidence, which often reduces road and infrastructure safety, thus posing potential dangers to road users [1,2]. The manual detection and repair of defective manhole covers via traditional methods can no longer meet the demands of modern cities in terms of timeliness, safety, and inspection accuracy. The automation of urban manhole-cover management, which acquires accurate location and ownership information—and their inspection and maintenance at higher operational safety levels—is now a primary focus in intelligent road administrations. The recent emergence and rapid advancement of mobile mapping [1,2,3] present effective methods for the monitoring and maintenance of defective manhole covers, while also functioning as data sources. The mobile mapping system is a new-generation multi-platform and multi-band mobile information acquisition system, this system is mounted on a carrier vehicle and integrated with various sensors, such as a laser scanner, inertial navigation system, global navigation and positioning system, and digital camera [4,5,6,7,8,9,10,11,12,13]. It offers a high-precision and high-efficiency means of acquiring large quantities of three-dimensional (3D) point cloud and image data in real-world environments, and has now become an indispensable component in the evolution of digital cities, providing long-term support in promoting the construction and management of modern cities and the transformation to digital management and operation. It also presents new possibilities and methods for the automated location, extraction, and identification of urban manhole covers.

Few studies have been conducted to date that address the monitoring and maintenance of urban manhole covers. Nan et al. [14] developed an underground practical sensing system to detect manholes beneath the urban pavement environment. Fu et al. [15] developed an intelligent system for manhole cover detection and management via the deployment of many sensors at each manhole cover to provide real-time monitoring. However, this method requires a considerable (and constantly increasing) amount of equipment to monitor all the manhole covers in a given urban area. Timofte et al. [16] presented a multi-view scheme for manhole detection and recognition based on a van-mounted camera and global positioning system (GPS) for data acquisition, but this method was limited in its ability to detect manhole covers, and the ownership information could not be obtained due to the low image resolution. It is also slow and inconvenient to monitor all the manhole covers via radio-frequency identification tagging [17]. Murasaki et al. [18] estimated the degree of manhole cover wear based on a texture recognition approach that used the local binary pattern feature of the image-processing method. Ji et al. [19] presented a manhole detection method that employed a multi-view matching and feature extraction technique based on close-range images, and inertial navigation system and light detection and ranging (LiDAR) data, and utilized canny edge detection for manhole cover recognition. However, this method depends on the detected edge of the manhole cover, which may be difficult to detect due to the similar intensities of the manhole cover and road. Yu et al. [2] used multi-scale tensor voting and distance thresholds to extract the manholes from two-dimensional georeferenced-intensity feature images [1], with these feature maps generated from mobile LiDAR data, but this method is computationally intensive. Yu et al. [20] detected manhole covers by using a supervised deep-learning model from the georeferenced-intensity feature maps that were generated via the interpolation of inverse-distance-weighted laser points. This method yielded better manhole-cover detection results, but it is solely based on laser points from the RIEGL VMX-450 mobile laser scanning system. Therefore, detailed cover ownership information could not be acquired, and the method only focused on manhole cover detection, with no manhole identification or assessment information obtained.

Two problems are identified in the above manhole-cover detection methods. First, the data obtained by conventional mobile-mapping systems [1,2,20] are not high-definition ground images, and therefore cannot provide detailed manhole-cover ownership information (e.g., ownership unit, use, and cover specifications), which is necessary for road administration purposes. Second, most researchers determine the manhole cover locations by using point cloud or image data, whereas the combined implementation of both data sources achieves higher precision results. Furthermore, no manhole cover assessments have been conducted during the detection and identification process to assess whether a given manhole cover needs to be repaired or replaced. In summary, current conventional mobile-mapping systems do not meet the growing demands of manhole cover detection, identification, maintenance, and management for urban road administration purposes.

This study proposes a modified manhole-cover detection, recognition, and assessment system that utilizes the integration of two image-acquisition devices. An improved sequential exposure scheme using a symmetrical arrangement of multiple cameras, in combination with a high-density laser scanner, is proposed to obtain high-density point cloud and ultra-high-resolution ground images simultaneously. An improved method based on the histogram of oriented gradients (HOG) descriptor with symmetry features, support vector machine (SVM), and statistical analysis is proposed. This improved and optimized system, in combination with the proposed data-processing method, enables the efficient and rapid detection, identification, and assessment of manhole covers. In this paper, we first provide a description of the developed manhole-cover detection system, and then introduce the key technologies used in manhole cover detection and maintenance, followed by an analysis of the experimental results.

## 2. Materials and Methods

### 2.1. Key System Optimization Technologies

**Figure 1:**
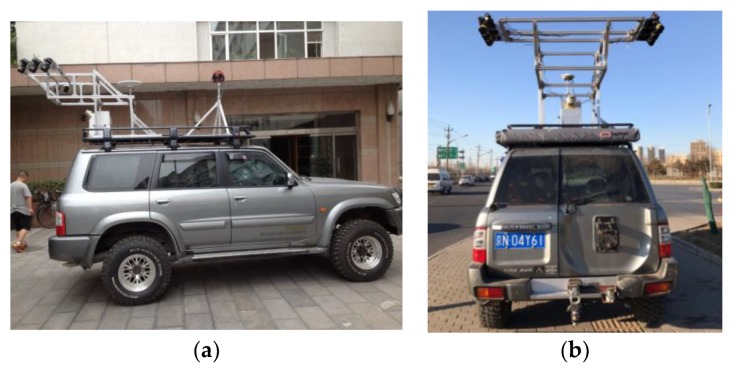
Modified mobile-mapping system. (**a**) Side view. (**b**) Back view.

**Figure 2:**
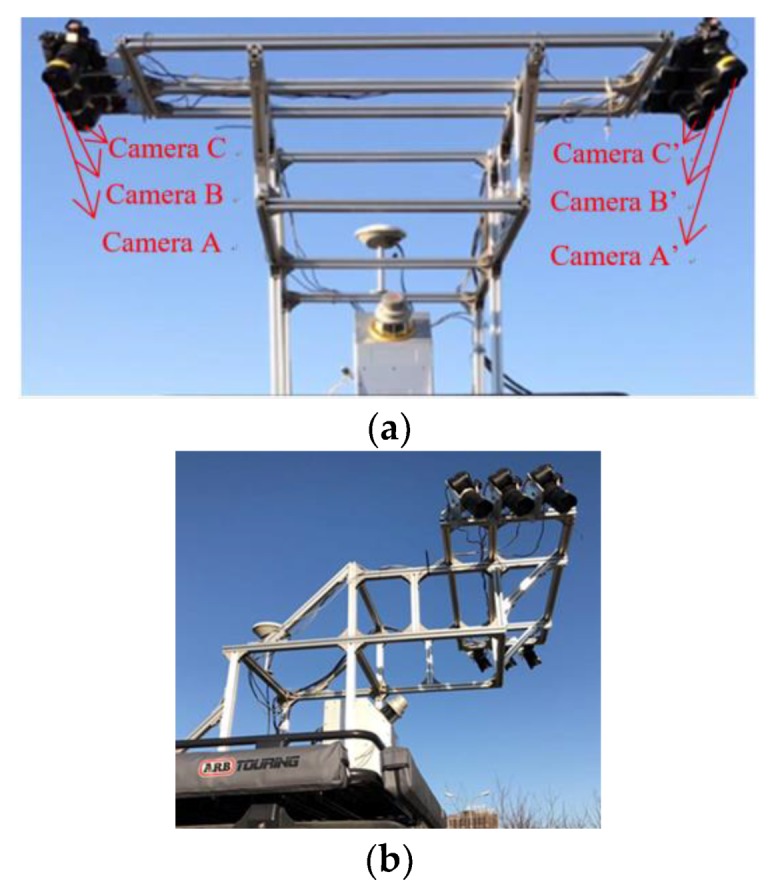
Structure of the reconstructed camera system and mounting frame. (**a**) Bottom view of the modified system. (**b**) Side view of the modified system.

The modified mobile-mapping system for the customized and professional detection and identification of manhole covers, here termed the SSW-D Mobile LiDAR System, is shown in Figure 1. It is a modification of the SSW mobile-mapping system [3], including the acquisition of high-resolution images and high-precision laser data, the key steps in the modification process are the equipment model selection and system reconstruction, which are detailed below: 

(1) Equipment selection: The sensor system needs to be constructed using the optimal vehicle, camera, and laser model. The vehicle is selected based on three factors: interior space, roof stability, and height. Full-sized sports utility vehicles or multi-purpose vehicles are the best choices due to their spacious interiors, which facilitates the storage of additional equipment, such as generators. The roofs of these vehicle models are also stable after the camera mount installation, and the extra height of these vehicles provides a broader field of view during acquisition. Mobile mapping systems are often equipped with panoramic or area array cameras, but these devices are unable to capture high-precision ground images. The Sony A7 camera with a 14-mm fisheye lens is chosen as the ideal camera for the system, and the Canon 5D II series camera is chosen as an alternative candidate, with the latter camera being a little larger, heavier, and more unstable to return to signal stability than the former camera. Road maintenance and repair is required when a manhole cover subsidence of 2 cm or more is detected. For higher accurate detection, we choose RIEGL laser scanner to acquire high-precision and high-density laser data, so as to assessment manhole cover.

(2) System construction and installation: A camera mount that extends from the rear of the vehicle is designed for the high-resolution ground image acquisition. The cameras are also tilted 20° toward the rear of the vehicle to increase the shooting range, as shown in Figure 2a,b. The Sony A7 manual states that the camera has a shooting speed of 1 image/s at the highest resolution, but the exposure interval is longer than one second during acquisition to prevent image loss. Furthermore, an image acquisition rate of 1 image/s requires a vehicle speed of 18 km/h, which seriously affects local traffic and the efficiency of our work. A sequential exposure method that uses multiple cameras in a symmetrical arrangement is therefore proposed. Three pairs of cameras (labeled A, B, and C in Figure 2a) are employed to acquire ground images sequentially, and they are positioned based on the load-bearing capability of the mounting frame, system stability, operation efficiency, image accuracy, and image overlap. The modified system is shown in Figure 2. The camera exposure is based on the mileage value, with the exposure signal triggered at a certain distance interval. This setup ensures that the data requirements are met for a vehicle speed of 40 km/h.

**Figure 3:**
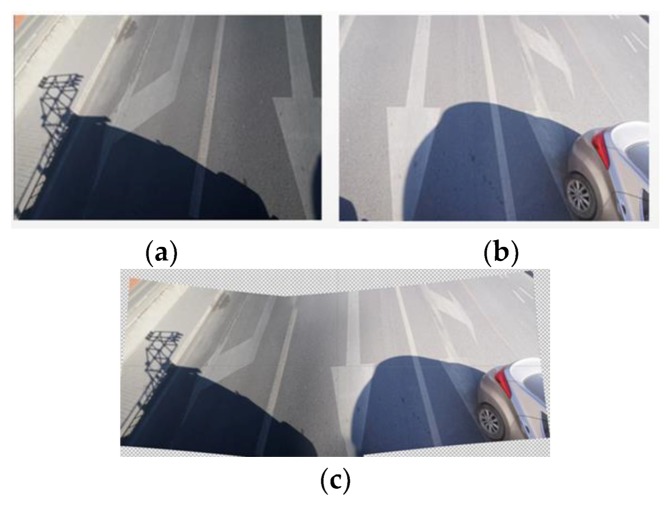
Obtained and fused ground images. (**a**) Ground image obtained by the left cameras. (**b**) Ground image obtained by the right cameras. (**c**) Fused ground image.

Symmetric exposure from both the left and right also increases the ground shooting range to three traffic lanes, as shown in Figure 3. Figure 3a,b are the ground images taken by the left and right cameras, respectively, and Figure 3c is the fused image, which yields an image that covers three lanes.

**Figure 4:**
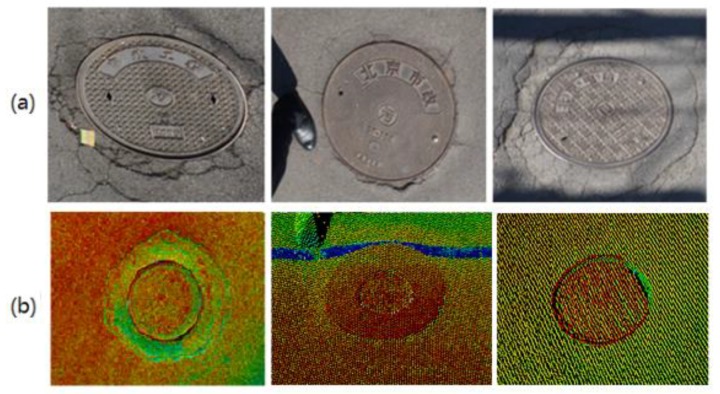
Example manhole-cover images. (**a**) Ground images. (**b**) Laser data images.

The above modifications are sufficient for the daytime detection, identification, and maintenance of manhole covers. The ownership information extracted from the manhole covers is clear and visible, as shown in Figure 4. Example ground images are shown in Figure 4a, and example LiDAR data from different manhole covers are shown in Figure 4b. The high-precision images and LiDAR data acquired by the improved SSW-D system provide a favorable database for the later detection and maintenance of manhole covers.

### 2.2. Key Detection and Identification Technologies

**Figure 5:**
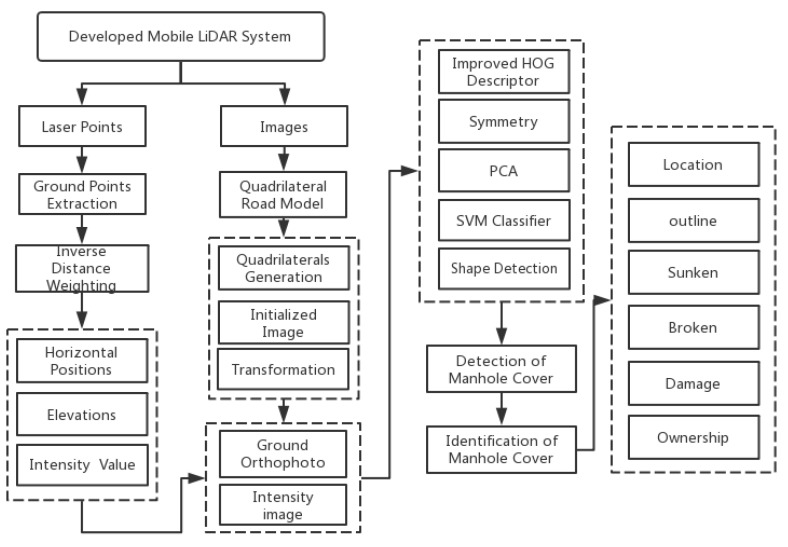
Framework for manhole cover detection and identification.

High-resolution images and high-density laser data of the road surface are acquired via the SSW-D system after sensor calibration. Images can provide high-resolution texture information, and laser data can provide high-precision 3D location information. However, there is no method to extract manhole cover images based on the combination of laser data and images simultaneously. As described in Section 1, it is difficult to apply current methods to the practical engineering of well cover detection, and solve the problems currently faced. In this paper, an automatic manhole-cover extraction method based on the combination of images and laser data is proposed; the two datasets are integrated in this study to utilize the strengths of both datasets and obtain more accurate cover-detection results. The key technology comprises four main components: (1) the generation of intensity images and (2) ground orthophotos, (3) manhole cover extraction and detection, and (4) manhole cover identification. This process is illustrated in Figure 5.

#### 2.2.1. Generation of Intensity Images

The manhole-cover detection method primarily involves an image-processing method that requires the conversion of 3D laser-scanned point cloud data into intensity-based images. Accordingly, the intensity-based images are generated from the ground laser points according to our previous study [3], where the ground points are first extracted based on the fluctuation trend in the elevation differences between the current laser point and the neighboring laser points of the adjacent scan lines. The laser point is considered as the ground point if the trend value is below a certain threshold. We then use these derived ground points to project the laser-scanned point data onto the XOY plane and rasterize the dataset based on the horizontal positions, elevations, and intensity information of laser data. 

This requires the range of the 3D point cloud ([$X_{min}$, $X_{max}$], [$Y_{min}$, $Y_{max}$], [$Z_{min}$, $Z_{max}$]) to be determined. The grid size (W× H) is calculated via Equation (1):

(1) $$\{\begin{array}{c} W=\left(X_{max}-X_{min}\right)/GSD\\ H=\left(Y_{max}-Y_{min}\right)/GSD \end{array},$$

where W is the width of the grid, H is the height of the grid, and GSD is the ground sample distance. The gray value of each grid cell (*i*,*j*) is determined by the relevant features of the laser-scanned points in the grid, such as their horizontal positions, elevations, and intensity values. The effect of the horizontal position is computed via inverse distance weighting, with larger weights assigned to the horizontal positions that are farther from the grid center and vice versa. The elevation is related to the mean difference of the elevation, with larger weights assigned to the elevations that possess smaller mean differences and vice versa. The gray value of each grid cell (*i*,*j*), $F_{ij}$ is then computed as:

(2) $$F_{ij}=\sum_{k=0}^{n_{ij}}(w_{ijk}\cdot I_{ijk})∕\sum_{k=0}^{n_{ij}}w_{ijk},$$

where $I_{ijk}$ is the intensity of the *k-th* laser-scanned point in the grid cell, $n_{ij}$ is the number of scanned points in the grid cell, and $w_{ijk}$ is the calculated weight of the *k-th* laser-scanned point, which is defined as:

(3) $$w_{ijk}=\alpha \times \left(\frac{\sqrt{2}GSD}{D_{ij}^{k}}\right)+\beta \times \frac{\left(h_{min_{ij}}-Z_{min}\right)}{(\left(Z_{max}-h_{max_{ij}}\right)\left(Z_{ij}^{k}-Z_{mean}\right))}$$

where α and β are the weight coefficients for the horizontal position and elevation, respectively, $D_{ij}^{k}$ is the distance between k laser point in grid cell (*i*,*j*) and its center. $h_{max_{ij}}$, $h_{min_{ij}}$, and $Z_{mean}$ are the maximum, minimum, and average elevations of the laser-scanned points in the grid cell, respectively, and $Z_{ij}^{k}$ is the elevation of the *k-th* laser-scanned point in the grid cell. The gray value of each grid cell (*i*,*j*) is obtained via Equation (2). Because the gray value range for the entire gridded area of the intensity image is $\left[I_{min},I_{max}\right]$ we therefore employ the normalized method to unify the gray interval over [0, 255]. These generated intensity images then become the data source for the manhole cover detection and identification.

#### 2.2.2. Generation of Ground Orthophotos

**Figure 6:**
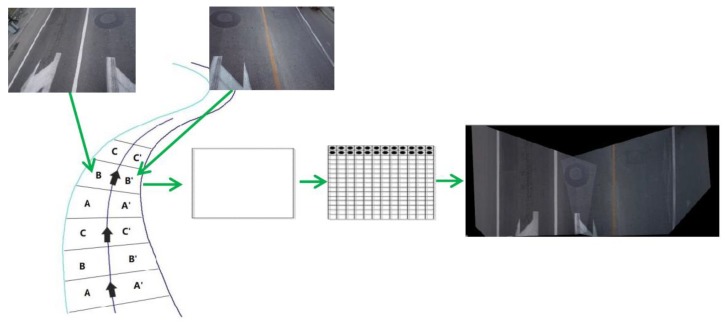
Schematic for the generation of the ground orthophotos.

There are additional difficulties in extracting the manhole cover location from the images acquired by the SSW-D system due to the varying degrees of manhole cover deformation. An improved quadrilateral orthographic road (IQOR) model is proposed for the generation of ground orthophotos to restore the true shape of the manhole covers. The IQOR model constructed in this study possesses a higher geometric accuracy because it is based on the image and laser points after registration synchronization to obtain the real road surface, instead of simplifying the pavement to a pure plane via inverse perspective mapping [21]. Quadrilateral generation, image initialization, and image transformation are the key steps in the IQOR model (Figure 6). 

The ground orthophoto process is as follows: A three-lane road strip is recreated by first extending each 1.5-lane image to both sides of the trajectory line based on the camera exposure mileage value to segment a given road strip, and then forming a series of closed quadrilateral blocks, with the z-value of the quadrilateral vertexes confirmed from the ground points. Image initialization takes a blank image and rasterizes the quadrilateral blocks according to the specified ground resolution, with the ground orthophoto resolution calculated from the focal length, image size, and height of the road surface. The initialized image is projected on a XOY plane on the road surface. The space transformation of the camera center and the initialized image are analyzed to obtain the RGB information based on the position and attitude information of the synchronized image at the same exposure time, which yield the RGB information from the intersection position of the transformed camera center and the location of the intersection of the initialized image and the synchronized image. The RGB value of each pixel is then retrieved to construct the ground orthophoto, as shown in Figure 6, which also illustrates the ground-orthophoto generation process. Each ground orthophoto image possesses a certain geographical reference from which the corresponding spatial position and RGB information can be obtained.

#### 2.2.3. Manhole Cover Detection

The rapid extraction and location of manhole covers are conducted using the generated intensity-based images and ground orthophotos. In this paper, an improved method based on an improved HOG descriptor, principal component analysis (PCA), symmetry characteristic, and shape detection to detect manhole covers is proposed. The improved HOG descriptor [22,23,24] and PCA method are used to reduce the image dimensions and the SVM is applied as a discrimination method. In addition, symmetry is introduced into the manhole-cover extraction method. Because the manhole cover itself has some circular or rectangle shape and symmetrical characteristics, the combination of the HOG descriptor and symmetrical features can achieve more effective manhole-cover filtering extraction. Symmetry features are obtained directly based on HOG descriptors. Symmetry descriptors are illustrated by the channel with eight bins of HOG descriptors. For a circular manhole cover, the upper ($C_{u}$), down ($C_{d}$), left ($C_{l}$), and right ($C_{r}$) sectors are symmetrically distributed, so the eigenvectors of the left $P_{u}^{l}$ and right regions $P_{u}^{r}$ are shown in Equation (4), and the adjusted symmetrical eigenvectors $P_{u}^{R^{\prime }}$ are shown in Equation (5).

(4) $$\begin{array}{ll} P_{u}^{l} & =[p_{\mathrm{ul}1}\;p_{\mathrm{ul}2}\;p_{\mathrm{ul}3}\;p_{\mathrm{ul}4}\;p_{\mathrm{ul}5}\;p_{\mathrm{ul}6}\;p_{\mathrm{ul}7}\;p_{\mathrm{ul}8}{]}^{T}\\ & =[p_{\mathrm{ur}1}\;p_{\mathrm{ur}2}\;p_{\mathrm{ur}3}\;p_{\mathrm{ur}4}\;p_{\mathrm{ur}5}\;p_{\mathrm{ur}6}\;p_{\mathrm{ur}7}\;p_{\mathrm{ur}8}{]}^{T} \end{array}$$

(5) $$\begin{array}{ll} P_{u}^{R^{\prime }} & =[p_{\mathrm{ur}1^{\prime }}\;p_{\mathrm{ur}2^{\prime }}\;p_{\mathrm{ur}3^{\prime }}\;p_{\mathrm{ur}4^{\prime }}\;p_{\mathrm{ur}5^{\prime }}\;p_{\mathrm{ur}6^{\prime }}\;p_{\mathrm{ur}7^{\prime }}\;p_{\mathrm{ur}8^{\prime }}{]}^{T}\\ & =[p_{\mathrm{ur}5}\;p_{\mathrm{ur}4}\;p_{\mathrm{ur}3}\;p_{\mathrm{ur}2}\;p_{\mathrm{ur}1}\;p_{\mathrm{ur}8}\;p_{\mathrm{ur}7}\;p_{\mathrm{ur}6}{]}^{T} \end{array}$$

The symmetric vector of $P_{u}^{l}$ and $P_{u}^{r}$ is defined by Equation (6), and $C_{u}={\left[\begin{array}{cccccccc} C_{u1} & C_{u2} & C_{u3} & C_{u4} & C_{u5} & C_{u6} & C_{u7} & C_{u8} \end{array}\right]}^{T}$, j is located at the range of [1,8], $C_{uj}$ is calculated based on Equation (6).

(6) $$C_{uj}=\{\begin{array}{cc} \frac{p_{uli}}{\sum_{k=1}^{8}p_{ulk}}/\frac{p_{uri}}{\sum_{k=1}^{8}p_{urk}}, & if\frac{p_{uri}}{\sum_{k=1}^{8}p_{urk}}>\frac{p_{uli}}{\sum_{k=1}^{8}p_{ulk}}\\ \frac{p_{uri}}{\sum_{k=1}^{8}p_{urk}}/\frac{p_{uli}}{\sum_{k=1}^{8}p_{ulk}}, & if\frac{p_{uri}}{\sum_{k=1}^{8}p_{urk}}\geq \frac{p_{uli}}{\sum_{k=1}^{8}p_{ulk}} \end{array}$$

Among them, the value range of elements in the symmetric vector $C_{u}$ is between 0 and 1, and the corresponding symmetric vector $C_{d}$ can also be obtained by choosing the similar principle. Due to the symmetric feature of the well cover, the symmetry vector itself has better symmetry. Then, the optimized HOG descriptor consists of the HOG descriptor’s own vector and the circular symmetry vector. However, the optimized HOG descriptor is high dimensions, and it contains excessive unnecessary redundant data, which not only directly causes computational pressure and increases the computational complexity, but also directly reduces the extraction accuracy. Accordingly, PCA was introduced in the proposed method. The PCA method is applied here to convert the original variables into a series of new and unrelated variables via an orthogonal transformation. A small number of new vectors are selected to represent the best original vectors and a function is defined by the new feature descriptors based on the actual need of the image, which reduces the image dimensions and speeds up the calculation. The last step consists of placing the feature descriptors of the sample data after PCA processing into the SVM classifier to carry out training. Detectors are also applied to the intensity or orthophoto images to generate negative and hard examples, which are used with the previously acquired features that train the final detectors. The obtained detectors are then placed into the SVM classifier [25] for the rapid location and automated extraction of manhole covers. 

This method can be used to extract manhole covers from both the intensity images and ground orthophotos. Manhole cover information is extracted from both datasets to demonstrate their respective advantages, with the final extraction result based on the final information that possesses higher accuracy.

#### 2.2.4. Manhole Cover Identification

**Figure 7:**
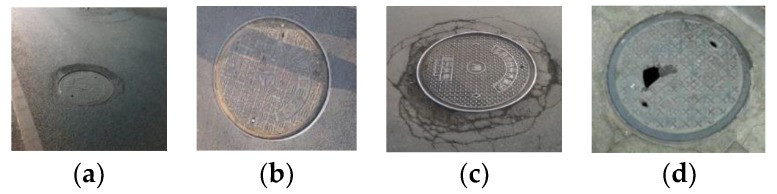
Example manhole cover conditions. (**a**) Sunken manhole rim. (**b**) Sunken manhole cover. (**c**) Damage to the periphery of the manhole. (**d**) Damaged manhole cover.

Manhole cover identification includes the acquisition of manhole maintenance and ownership information. Manhole cover maintenance is primarily used to determine if a manhole cover is defective, and if maintenance or replacement is necessary. Defective manhole covers include sinking manhole covers, manhole ring-height differences, broken manhole covers, and damage to the periphery of manhole covers, which is shown in Figure 7. For example, a height difference of greater than 2 cm between the manhole cover and manhole ring is indicative of a sinking well cover, which requires maintenance. Damage to the periphery of manholes is evident when the road surface around the manhole shows clear signs of damage, with an accompanying large height fluctuation observed. Manhole cover damage is any breakage of the manhole cover, which indicates an incomplete or insecure cover. Therefore, manhole cover maintenance is primarily achieved by evaluating the heights of the cover, rim, and periphery of the rim, which is based on the height difference values between the manhole cover, rim, and periphery of the rim, to determine if a height-difference threshold value is exceeded. Manhole ownership information consists of the ownership unit, usage (e.g., water and sewage discharge, electricity and gas supply, and communication), and cover specifications. Cover diameters can be identified as either 60 or 80 cm when the specifications are obtained by their location and outline. However, the ownership unit and usage must be identified from the ground images. Manhole-cover outline detection is therefore the key to inspecting manhole cover conditions.

A graph-based image segmentation method, OneCut [26,27], is employed to delineate the manhole cover outlines by using the location information obtained from the intensity-based images and ground orthophotos. OneCut is an improved image-splitting algorithm by Tang et al. [27] that is based on GrapCut. It uses a fast globally optimized binary partitioning technique, and requires a priori specifications of a rectangle framed around the objects of interest. This frame contains the foreground and some background information. Pixels that fall outside the frame are hard-constrained as background pixels. The minimum energy function for image partitioning is given by Equation (7).

(7) $$E\left(S\right)=\left|\overset{-}{S}\cap^{\text{​}}R\right|-\beta ||\theta^{S}-\theta^{\overset{-}{S}}||{}_{L1}+\lambda \left|\partial S\right|,$$

where *S* and $\overset{-}{S}$ represent the foreground and background images to be segmented, respectively, *R* is the bounding box, $\;\theta^{S}$ and $\theta^{\overset{-}{S}}$ are unnormalized histograms of the foreground and background, respectively, and the value of  λ is based on experiments.  |∂S| is the contrast-sensitive smoothness term, which is calculated from Equations (8) and (9):

(8) $$\left|\partial S\right|=\sum^{\text{​}}\left(\frac{1}{\left||p-q|\right|}\cdot e^{\frac{-||I_{p}-I_{q}||{}^{2}}{2\sigma^{2}}}\cdot \left|S_{p}-S_{q}\right|\right)$$

(9) $$\sigma^{2}=\frac{1}{N_{n}}\underset{\left(p,q\right)\in n\left(I\right)}{\sum }||I_{p}-I_{q}||{}^{2}$$

where {*p*,*q*} represents the case of unordered pairs of neighbors, $N_{n}$ is the number of elements in n(I), and n(I) is a set of pairs of adjacent pixels in image I. The above model requires the designation of a rectangular frame that contains the objects of interest. Here, seed points are provided in the actual computation to replace the rectangular frame. The location of the manhole cover and the region of interest (ROI) containing the cover can be obtained as outlined in Section 2.2.3. The pixels that define the outline of the cover are hard-constrained as background pixels, and the pixels within a certain distance from the center of the cover are hard-constrained as foreground pixels based on the ROI. This removes the rectangular frame requirement of the above model, which then yields the optimized minimum energy function as:

(10) $$E_{seed}\left(S\right)=-\beta ||\theta^{S}-\theta^{\bar{S}}||{}_{L1}+\lambda \left|\partial S\right|,$$

where *β* is defined as:

(11) $$\beta =\beta_{img}=\frac{\left|R\right|}{-||\theta^{R}-\theta^{\bar{R}}||{}_{L1}+\left|\Omega \right|/2}\cdot \beta^{\prime },$$

and $\beta^{\prime }$ is a global parameter that is generally assigned a value of 0.9.

Splitting is conducted based on this energy function (Equation (10)) to produce a binary image, and the best-fit circle that represents the manhole cover is then obtained. The cover diameter and specifications can be obtained from the cover outline, as well as the corresponding point clouds and image data. 

**Figure 8:**
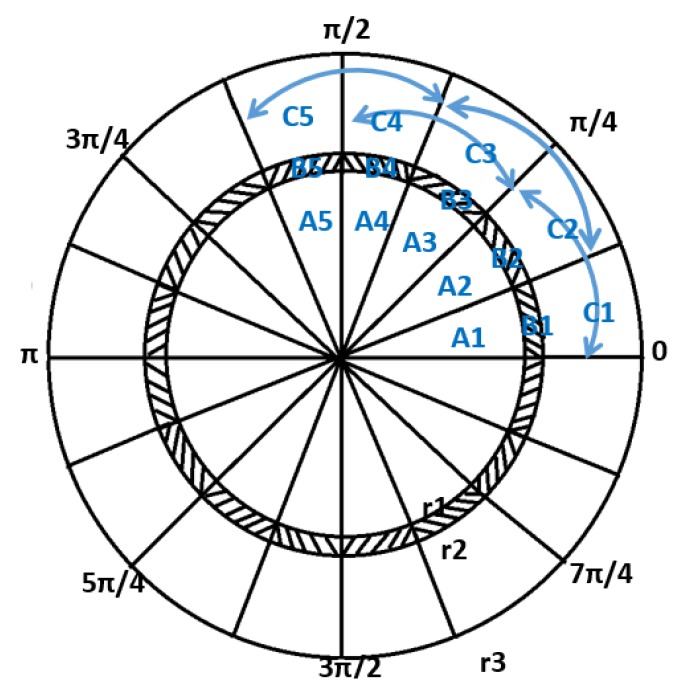
Sector decomposition diagram for manhole maintenance analysis.

The criteria for judgment are shown in Equation (12) based on Figure 8. The manhole covers are divided into 16 areas. In order to judge whether the covers are manhole cover damaged, subsidence or surround damage, we mainly rely on the laser point cloud data in the adjacent 45-degree fan area. As shown in the figure, A1, B1 and C1, respectively, represent the manhole cover, the manhole outer ring and the surrounding area of the manhole covers. r1, r2 and r3 are represent the radii of the manhole cover, the manhole outer ring and the surrounding area of the manhole covers. Statistical analysis is used to assess the height difference of the area surrounding the cover. The periphery of a manhole is classified as damaged if the height difference fluctuation exceeds a certain threshold, with the height difference between the manhole cover and its surroundings used to identify the degree of manhole cover subsidence. Other geometric parameters can also be evaluated in the same way using the laser-scanned point clouds. The criteria for judgment are shown in Equation (12).

(12) $$\{\begin{array}{c} \Delta f_{gc}^{i}=\left|{\bar{Z}}_{A_{i}+A_{i+1}}-{\bar{Z}}_{B_{i}+B_{i+1}}\right|\\ \Delta f_{xc}^{i}=\left|{\bar{Z}}_{A_{i}+A_{i+1}}-{\bar{Z}}_{C_{i}+C_{i+1}}\right|\\ \Delta f_{ps}^{i}=\left|\overset{n}{\underset{i=1}{\sum }}Z_{A_{i}}/n-{\bar{Z}}_{A_{i+}+A_{i+1}}\right|\\ \Delta f_{zbps}^{i}=\left|\overset{n}{\underset{i=1}{\sum }}Z_{C_{i}}/n-{\bar{Z}}_{C_{i+}+C_{i+1}}\right| \end{array}$$

where $\Delta f_{gc}^{i}$ represents the height difference of the sector area in the i-th block, $\Delta f_{xc}^{i}$ represents the subsidence value of the sector area in the i-th block, $\Delta f_{ps}^{i}$ represents the cover damage value in the sector area in the *i-th* block, and $\Delta f_{zbps}^{i}$ denotes the periphery damage value in the sector area in the *i-th* block. If the above calculation threshold exceeds the evaluation value of the defect manhole cover, it is considered that there is a disease in the cover. The accuracy of the results is directly related to the laser data density. The modified system in Section 2 ensures the high-performance efficiency and accuracy of the data during acquisition. Corresponding images of the manhole cover site are obtained and high-definition images of the cover are also acquired in a semi-automated manner to obtain manhole-cover ownership information (i.e., extraction of the cover inscriptions). Manhole cover detection and identification via a mobile-mapping system transfers a large amount of tedious outdoor work to the office, while also supplying high-precision and detailed data for manhole cover maintenance. 

Our developed system and proposed method of the detection, identification and assessment method transform the operational mode of road maintenance, and provides technical support. 

## 3. Results and Discussion

### 3.1. Experimental Data

The experimental datasets are acquired using the SSW-D Mobile LiDAR System described in Section 2 to verify the feasibility, practicality, and effectiveness of the proposed method. The SSW-D is integrated with a RIEGL VUX-1HA laser sensor, cameras (SONY A7), an inertial measurement unit, GPS antenna, odometer, and other features, with the specific sensor parameters listed in Table 1. A RIEGL VUX-1HA laser sensor with the following specifications is used in the customized mobile-scanning system mentioned: laser pulse repetition-rate = 1014 kHz, scan frequency (number of laser lines recorded per second) = 250 Hz, and measuring range = 2–200 m at a reflectivity of 80%. A SONY A7 camera, with a focal length of 12 mm, pixel size of 6 μm, and image size of 24 million pixels, is used. The POS2010 inertial navigation system (Beijing) [3] is used.

**Figure 9:**
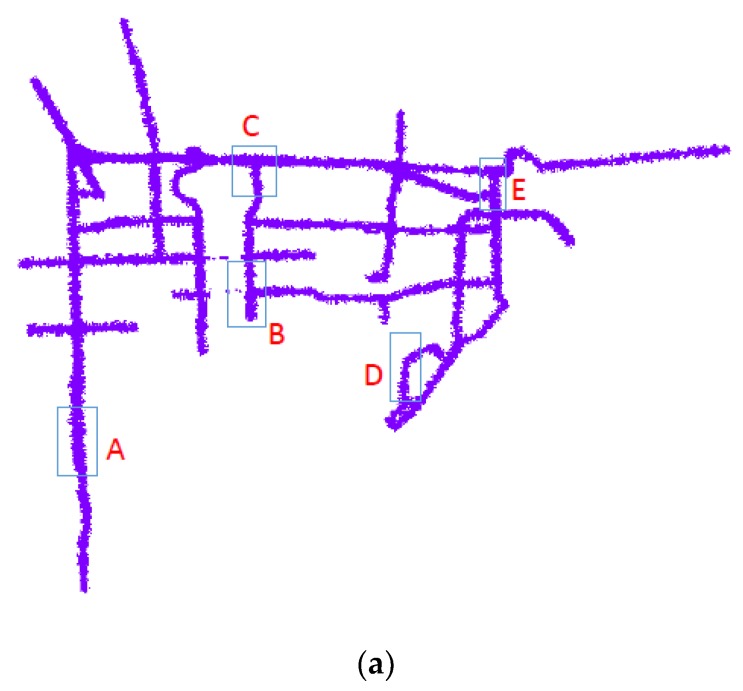

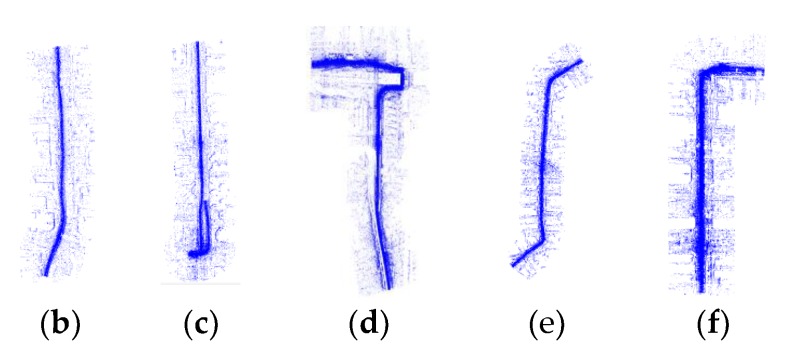
Schematic representation of the study area and the five road-section samples. (**a**) The entire study area. (**b**) Sample A. (**c**) Sample B. (**d**) Sample C. (**e**) Sample D. (**f**) Sample E.

For convenience, we choose an urban road in Beijing, China, with more manhole covers, as the experimental area to verify the effectiveness and feasibility of the proposed method. The vehicle is driven at 40 km/h during the data acquisition, with the entire study area shown in Figure 9a, among, Figure 9b–f is the sample A,B,C,D,E located at the area of Figure 9a. A total of 216.885 km of roads were surveyed, with 1.80 × 10^5^ high-definition ground images and 6016 laser files acquired. The laser files contain 1.36 × 10^9^ laser-scanned points at a point density of 1800 p/m^2^. The data include various complex ground features, such as vehicles, trees, buildings, and acoustic barriers, to meet road administration requirements, and ensure effective and accurate manhole cover detection. The following experimental analyses are performed on the five road-section samples. The data acquired with the proposed system in this study are used for the automated extraction of manhole covers, and the subsequent detection, identification, and assessment of manhole covers employ the proposed method. This manhole cover experiment confirms the feasibility of the acquisition system and the effectiveness of the method.

### 3.2. Manhole Cover Detection

Ground point extraction is the first step in manhole cover detection from laser-scanned point clouds. The effective selection of ground points and the elimination of non-ground points significantly reduce the computational workload of large laser datasets. Here, we directly use the ground points in the manhole cover detection and identification, with the ground point extraction based on the method outlined in our previous paper [3]. An image resolution of 0.01 m is set to generate the intensity-based images from the ground points. The modified HOG–SVM joint method with symmetry features used for the extraction in this study requires sample data for the calculation, which are created directly from the intensity images. The final intensity images include 13,838 positive samples and 10,846 negative samples. Five road-section samples are randomly selected in the study area to provide further details on the extraction results, as shown in Table 2. The first column shows the ground points, the second column data displays the manhole-cover detection result as green rectangles, the third column data shows the manhole-cover outline extraction result as blue circles, and the fourth column depicts the superimposed result based on the laser points. It can be seen that the proposed method effectively extracts the location of the manhole covers and their outline based on the intensity images generated from the laser points. 

**Figure 10:**
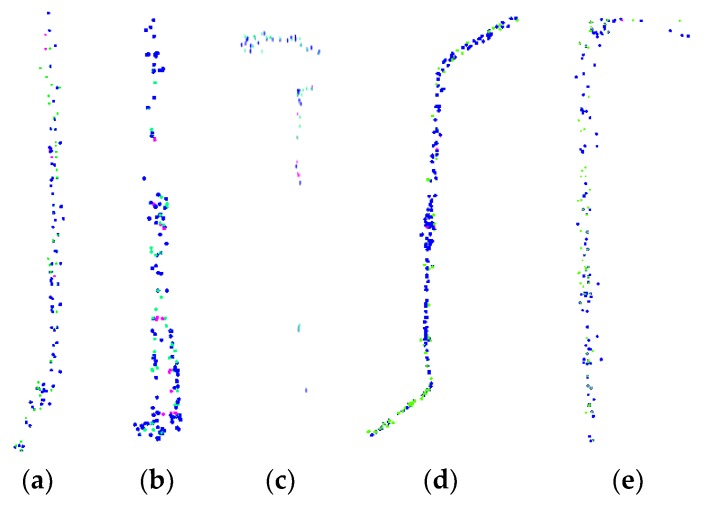
Detection results. (**a**) Sample A. (**b**) Sample B. (**c**) Sample C. (**d**) Sample D. (**e**) Sample E.

The manhole cover extraction from the images consists of first generating the ground orthophotos from the images, with the manhole cover extraction and detection carried out using the method described in the Section 2.2.3. The high degree of agreement between the laser-scanned point clouds and image data yields overlaps in the extraction results that need to be removed. In total, 7015 positive samples and 8185 negative samples are generated from the ground orthophotos. The robustness of the proposed method is verified by the data from road samples A–E, as shown in Figure 10. The blue points are the extraction results from the point clouds, the green points are the results from the image data, and the magenta points are the undetected covers. Numerous overlaps are present in the extracted structures detected by the two data sources, as seen in Figure 10. However, some manhole covers can only be extracted from the intensity-based images, while others can only be extracted from the ground orthophotos. Improved results are therefore achieved by merging the extraction outcomes of the two data sources and removing the repeated extracted results, as given in Table 3.

We computed the completeness (CPT), correctness (CRT), and F-measure [3] to assess the success of the extracted result via the proposed method against manual inspection. CPT indicates the percentage of detected manhole covers. CRT is the percentage of correct manhole covers detected. CPT = CA/TA, CRT = CA/TC, and F-measure = 2 × (CPT × CRT)/(CPT + CRT), where CA is the correct number of extracted manhole covers, TA is the total number of manhole covers via manual inspection, and TC is the total number of extracted manhole covers. These assessment results are compared with the previous method [28] based on the HOG descriptor without symmetry features, which extracted manhole covers by using laser points, with the results given in Table 4. 

By combining the laser points and images as outlined in the proposed method, the average CRT, CPT, and F-measure for manhole cover detection are 96.18%, 94.27%, and 95.22%, respectively. When compared with our previous method [28], which used only laser points, the CPT, CRT, and F-measure are all higher for the proposed method, which employs a combination of laser points and images. Although the proposed method obtained better extraction results, it failed to extract 5.37% of the manhole covers, the laser points incorrectly extracted 3.38% of the manhole covers, and the ground images incorrectly extracted 4.93% of the manhole covers. Four key factors likely influence these manhole-cover extraction failures: (1) the integrity of the data, as vehicles, pedestrians, and other obstructions cause unavoidable data loss in both the laser point and image acquisitions; (2) laser point density, which is related to the range, angle, long-term vehicle rolling, heat, weather, and other factors, with a higher density of laser points yielding a higher extracted result; and (3) interference from ground objects that are similar to the manhole covers (e.g., bicycle marks and wheels), as these objects can easily be mistaken for manhole covers. The following three measures can be incorporated to improve the extracted result. First, a vehicle speed limit should be enforced to avoid reducing the point cloud density due to a fast-travelling vehicle. Second, the data acquisition should be performed during the daytime during non-peak traffic hours to improve the work efficiency and integrity of the acquired data. Finally, the interference of similar ground objects can be addressed by either adding the negative samples containing these interfering features or employing a deep-learning method that participates in the combined laser point/ground image-based manhole cover extraction.

It can be concluded from the above discussion that the two data sources acquired with the modified mobile LiDAR system presented in this study and processed via the proposed method could effectively realize the automated detection of manhole covers to obtain a better extraction result. The complementary advantages of the two data sources can improve the accuracy and completeness of the results. Better extraction results would also validate the effectiveness and feasibility of the proposed modification scheme.

### 3.3. Manhole Cover Identification

**Figure 11:**
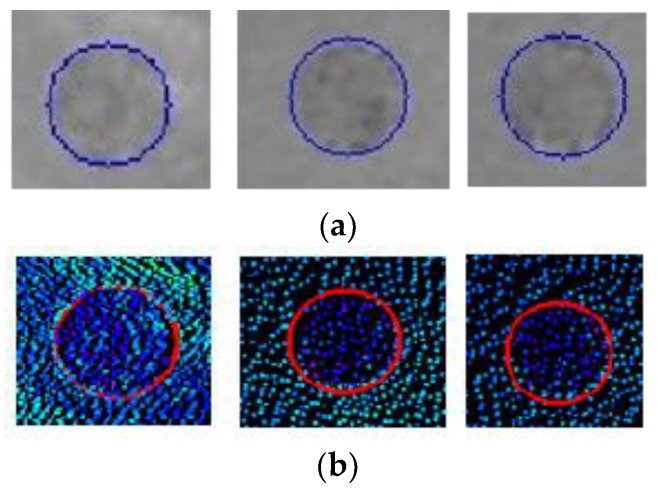
Detection of the manhole cover outline. (**a**) Ground image examples. (**b**) Laser data examples.

Manhole cover identification includes manhole maintenance and ownership information acquisition, which is achieved based on the extraction results presented in Section 2.2.4. Manhole cover maintenance is determined from the elevation fluctuations of the manhole cover, its rim, and the surrounding areas, which require the manhole cover outline. We can obtain the manhole cover outline via the proposed method, with the extracted outline result computed from the intensity-based images (Figure 11a) and determined by superimposing the computed outlines on the point cloud data (Figure 11b). 

The elevation fluctuations of the manhole cover, its rim, and the surrounding areas can be calculated based on the manhole cover outline to identify whether the manhole cover requires maintenance or replacement. Ownership information is also acquired based on the manhole location, with the aid of the real-time amplification function in the SWDY program, which has been developed by our team. Each real-time manhole-cover photo is obtained directly from its respective location to acquire the ownership information, which can also be used to verify the correctness of the test results. The manhole cover diameter is calculated from the outline data, where the diameter is generally either 60 or 80 cm. The number of covers with abnormal elevations in road sections A–E is determined by statistical analysis. Two damaged manhole covers, 10 sinking manhole covers, 13 sinking manhole rings, and 18 broken manhole covers, which comprise 0.29, 1.45, 1.88, and 2.61% of the total manholes on these five road sections, respectively, are identified. This indicates that 6.24% of the manholes require maintenance. The experimental result highlights the potential to conduct on-site maintenance directly. The proposed method enabled the identification of 10,399 manhole covers from the point-cloud laser data and 9759 from the ground image data for the entire experimental dataset. The manual detection of such a large number of covers would entail a heavy workload, high risk, and low detection accuracy.

The modified acquisition system presented in this study, and the corresponding manhole-cover detection method transforms the surveying, data processing, inspection, and maintenance of manhole covers into a primarily office-based endeavor that improves both work efficiency and detection accuracy, while also reducing the risk generally associated with road works. The manhole cover detection and identification procedure conducted in this study has the potential to improve the management and maintenance of manhole covers for road administration purposes. 

Therefore, the developed system and the proposed method are effective in the detection and identification of manhole covers. This system is able to acquire an abundance of ground data, including high-density, high-precision laser-scanned point clouds and high-resolution ground images. The manhole cover information extracted via the proposed method aids in the data collection, inquiry, inspection, and statistical analysis of resources for road administration purposes. Furthermore, our improved acquisition scheme and extraction method are not limited to the extraction of manhole covers, but can also be applied to other ground features, such as road markings and road edge boundary. 

Compared to traditional manual methods, this method improves the automation, efficiency, accuracy, and security. However, this method also has some limitations. For shaded manhole covers in grass, it is easy to be shaded in the acquisition of manhole cover, leading to the failure of extraction and identification of the well cover. This needs to be combined with manual detection methods.

## 4. Conclusions

A sequential exposure scheme using multiple cameras in a symmetric arrangement is proposed as a modification and upgrade to the original acquisition design for manhole cover detection. Careful equipment model selection and installation are undertaken to meet the requirements for the effective detection of manhole covers. High-density, high-precision laser-scanned point clouds and ultra-high-resolution ground images are acquired with this modified system, which also makes the extraction of manhole cover conditions and ownership information possible. Modified HOG and SVM algorithms are applied to the intensity-based ground images and orthophotos generated from point clouds and ground images, respectively, to obtain the location, ownership, and state-of-health information of the manhole covers. The detection average accuracy is 96.18%, data completeness is 94.27%, and F-measure is 95.22%. The method described in this paper is highly efficient, accurate, and safe. It has been practically applied, and is currently used on the roads of Beijing to inspect and analyze manhole covers thoroughly. This method provides strong technical support in the data acquisition, surveying, and inspection of manhole covers in road administration works, and also promotes the intelligent construction and management of urban infrastructure.

While satisfactory experimental results and verifications are obtained using the manhole cover detection and identification methodology presented here, there is still room for improvement in the following areas. Deep learning should be integrated into the detection and extraction of the manhole cover information to improve the extraction results further. Future research should also implement automatic manhole-cover identification, with a focus on any unique identifiers that are engraved on the covers. Furthermore, data collection during peak traffic times should be avoided to reduce shielding and data gaps.

## Figures and Tables

**Table 1:** Device specifications.

Device	Specifications		Device	Specifications	
RIEGL	Laser pulse repetition rate	1014 kHz	Camera	Focal length	12 mm
	Scan frequency	250 Hz		Pixel size	6 μ
	Range	2–200 m at a reflectivity of 80%		Number of pixels	24 million
	Echoing mode	Multi-echo		Maximum resolution	6000 × 4000
	Relative measurement accuracy	≤1 cm		Exposure interval	1 s
	Divergence	0.3 mrad	POS2010	Positioning accuracy	Roll: 2‰ Pitch: 2‰ Yaw: 5‰
	Field of view	360°			
	Operation temperature	−10 to 40 °C		Horizontal position accuracy	<10 cm
	Safety grade	II		Elevation accuracy	<5 cm

**Table 2:** Manhole cover detection results.

Ground Points	Cover Detection	Outline Detection	Superimposed Result
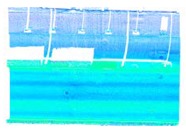	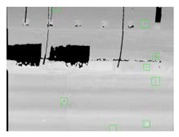	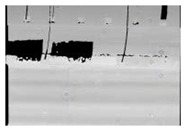	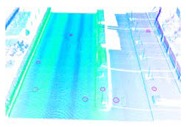
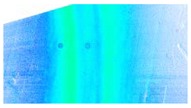	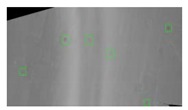	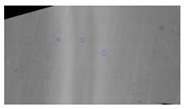	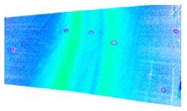
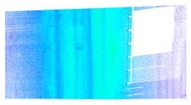	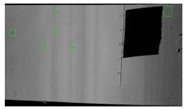	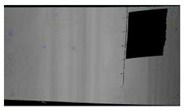	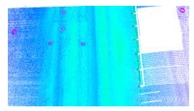
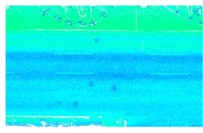	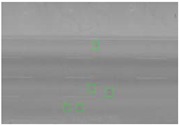	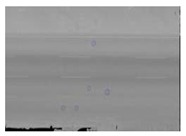	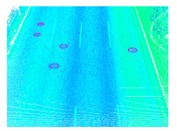
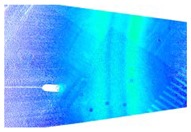	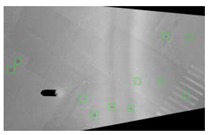	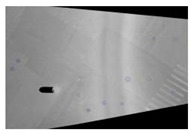	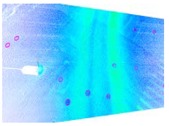

**Table 3:** Detection results from the laser data and ground images.

Zone	Total	Correct (Laser Data)	Correct (Images)	Failures (Laser Data)	Failures (Images)
A	122	104	108	6	8
B	155	130	135	4	7
C	63	55	58	3	4
D	210	174	190	6	9
E	139	115	105	4	6

**Table 4:** Manhole cover assessment result comparison between the previous method [20] and proposed method.

	Previous Method [28]			Proposed Method		
	CRT	CPT	F-Measure	CRT	CPT	F-Measure
A	0.9455	0.8525	0.8966	0.9487	0.9590	0.9538
B	0.9701	0.8387	0.8997	0.9720	0.9226	0.9467
C	0.9483	0.8730	0.9091	0.9483	0.9206	0.9342
D	0.9667	0.8286	0.8923	0.9703	0.9619	0.9661
E	0.9664	0.8273	0.8915	0.9697	0.9496	0.9595
AVG	0.9594	0.8440	0.8978	0.9618	0.9427	0.9522

## References

[1] Yu Y., Li J., Guan H., Wang C. Automated Detection of Road Manhole Covers from Mobile LiDAR Point-Clouds Based on a Marked Point Process. Proceedings of the Fifth International Conference on Geo-Information Technologies for Natural Disaster Management.

[2] Yu Y. (2014). Automated extraction of manhole covers using mobile lidar data. Remote Sens. Lett..

[3] Yang M.M., Wan Y.C., Liu X.L., Xu J.Z., Chen M.L., Sheng P. (2018). Laser data based automatic recognition and maintenance of road markings from MLS system. Opt. Laser Technol..

[4] Ma H., Pei Z.H., Wei Z.Y., Zhong R.F. Automatic Extraction of Road Markings from Mobile Laser Scanning Data. Proceedings of the International Archives of the Photogrammetry, Remote Sensing and Spatial Information Sciences Geospatial Week.

[5] Liu J.H. (2011). Research on algorithm of automatically recognizing and positioning road manhole covers based on vehicle-mounted sensors. Appl. Res. Comput..

[6] Yang B., Fang L., Li J. (2013). Semi-automated extraction and delineation of 3D roads of street scene from mobile laser scanning point clouds. ISPRS J. Photogramm. Remote Sens..

[7] Kumar P., Mcelhinney C.P., Lewis P. (2013). An automated algorithm for extracting road edges from terrestrial mobile LiDAR data. ISPRS J. Photogramm. Remote Sens..

[8] Guan H., Li J., Yu Y. (2014). Using mobile laser scanning data for automated extraction of road markings. ISPRS J. Photogramm. Remote Sens..

[9] Yan W.Y., Morsy S., Shaker A. (2016). Automatic extraction of highway light poles and towers from mobile LiDAR data. Opt. Laser Technol..

[10] Li L., Li D., Zhu H. (2016). A dual growing method for the automatic extraction of individual trees from mobile laser scanning data. ISPRS J. Photogramm. Remote Sens..

[11] Riveiro B., González-Jorge H., Martínez-Sánchez J. (2015). Automatic detection of zebra crossings from mobile LiDAR data. Opt. Laser Technol..

[12] Guo J., Tsai M.J., Han J.Y. (2015). Automatic reconstruction of road surface features by using terrestrial mobile lidar. Autom. Constr..

[13] Lindner L., Sergiyenko O., Rodríguez-Quiñonez J.C., Rivas-Lopez M., Hernandez-Balbuena D., Flores-Fuentes W., Natanael Murrieta-Rico F., Tyrsa V. (2016). Mobile robot vision system using continuous laser scanning for industrial application. Ind. Robot Int. J..

[14] Nan T., Mu X., Lin L. Manhole detection and location for urban pavement. Proceedings of the IEEE 2003 Intelligent Conference on Transportation Systems.

[15] Fu X. Manhole Cover Intelligent Detection and Management System. Proceedings of the International Conference on Electronic, Mechanical, Information and Management Society.

[16] Timofte R., Gool L.V. Multi-view manhole detection, recognition, and 3D localisation. Proceedings of the 2011 IEEE International Conference on Computer Vision Workshops.

[17] Chang A.Y., Yu C.S., Lin S.C., Chang Y.Y., Ho P.C. Search, Identification and Positioning of the Underground Manhole with RFID Ground Tag. Proceedings of the 2009 Fifth International Joint Conference on INC, IMS and IDC.

[18] Murasaki K., Sudo K., Taniguchi Y. (2015). Manhole cover wearing detection by photo-taking. Trans. Soc. Instrum. Control Eng..

[19] Ji S., Shi Y., Shi Z. (2012). Manhole cover detection using vehicle-based multi-sensor data. ISPRS Int. Arch. Photogramm. Remote Sens. Spat. Inf. Sci..

[20] Yu Y., Guan H., Ji Z. (2015). Automated detection of urban road manhole covers using mobile laser scanning data. IEEE Trans. Intell. Transp. Syst..

[21] Wang B.F., Qi Z.Q., Ma G.W. (2014). Robust lane recognition for structured road based on monocular vision. J. Beijing Inst. Technol..

[22] Kassani P.H., Teoh A.B.J. (2017). A new sparse model for traffic sign classification using soft histogram of oriented gradients. Appl. Soft Comput..

[23] Zhang L.H. (2013). Human Detection Based on SVM and Improved Histogram of Oriented Gradients. Appl. Mech. Mater..

[24] Said Y., Atri M., Tourki R. Human detection based on integral Histograms of Oriented Gradients and SVM. Proceedings of the International Conference on Communications, Computing and Control Applications.

[25] Flores-Fuentes W., Rivas-Lopez M., Sergiyenko O., Gonzalez-Navarro F.F., Rivera-Castillo J., Hernandez-Balbuena D., Rodríguez-Quiñonez J.C. (2014). Combined application of power spectrum centroid and support vector machines for measurement improvement in optical scanning systems. Signal Process..

[26] Rother C., Kolmogorov V., Blake A. Grabcut-interactive foreground extraction using iterated graph cuts. Proceedings of the ACM transactions on Graphics (SIGGRAPH).

[27] Tang M., Gorelick L., Veksler O. GrabCut in One Cut. Proceedings of the IEEE International Conference on Computer Vision.

[28] Yang M.M., Wan Y.C., Liu X.L., Yue G.J., Wang L.Z., Wei Z.Y. (2018). Automatic Location and Extraction of Manhole Based on Mobile LiDAR System. Chin. J. Lasers.

